# State Board of Health—The Law

**Published:** 1875-07

**Authors:** 


THE STATE BOARD OF HEALTH of Georgia has been organized during the past month, and measures taken to put the law creating the board into vigorous operation. For the information of our readers we republish the law, and also the constitution and organization of the board, the several committees, etc.

*An Act to create a State Board of Health for the protection of life and health, and to prevent the spread of diseases in the State of Georgia, and for other purposes.*

SECTION I. *The General Assembly of the State of Georgia do enact,* That within ninety days after the passage of this Act, the Governor shall appoint nine physicians, of skill and experience who shall have been regular graduates of medicine, and practitioners of not less than ten years, one from each Congressional District in the State, who, for the time being, shall be Sanitary Commissioners for the said Districts, and the said Sanitary Commissioners, together with the Comptroller General and Attorney

General and State Geologist, shall constitute a Board of Health, to be called the Board of Health of the State of Georgia, five members of which, at any regularly called or adjourned meeting, shall constitute a quorum for the transaction of business.

SEC. II. *Be it further enacted*, That the said nine physicians, so appointed, shall hold office as such Sanitary Commissioners, respectively, for the terms following, namely: Three for two years, three for four years, and three for six years, and until their successors are appointed and qualified. After the appointment of said nine physicians as aforesaid, they shall meet in the office of Secretary of State, upon notice from him of the day of meeting, and shall proceed, under his direction, to determine by lot which of them shall hold for the respective terms of two, four and six years, the said office of Sanitary Commissioners. Immediately, and before entering upon the duties of the office, they shall take the oath prescribed for State officers by the Constitution of the State, and shall file the same in the office of Secretary of State, who, upon receiving the said oath of office, shall issue to each of said Commissioners a certificate of appointment for his respective term of office, so determined as aforesaid; upon receiving which they shall severally be and become Sanitary Commissioners, and shall possess and exercise the powers, and perform the duties of said Board, as defined in this act.

SEC. III. *Be it further enacted*, That the term of office of said Sanitary Commissioners, after the expiration of the terms aforesaid, shall be six years, and they shall be appointed by the Governor. Any vacancies that may occur, by reason of death, removal from office or otherwise, shall be filled in like manner.

SEC. IV. *Be it further enacted*, That immediately after the nine appointed Sanitary Commissioners shall have taken the oath of office as above provided, they shall meet with the Attorney General, the Comptroller General and the State Geologist, and organize as a Board of Heath, by electing one of said Board to be President, and by appointing a proper person, who shall be a physician, to be Secretary of said Board, and the successive Presidents of said Board of Health shall be annually elected by said Board from the members thereof. The Secretary shall continue in office, as such, until removed, by the election of a successor, or otherwise, and shall be executive officer of said Board, and shall receive an annual salary not to exceed one thousand dollars. The Sanitary Commissioners shall receive no salary; but the actual personal expenses of any member, while engaged in the duties of the Board, shall be allowed and paid.

SEC. V. *Be it further enacted*, That said Board shall take cognizance of the interest of health and life among the people of the State; they shall make inquiries in respect to the causes of diseases, and especially of epidemics, and investigate the sources of mortality, and the effects of localities, employments, and other conditions upon the public health.

SEC. VI. *Be it further enacted*, That it shall be the duty of said Board to obtain, collect and preserve such information relating to deaths, diseases and health as may be useful in the discharge of its duties, and contribute to the promotion of health, or the security of life in the State of Georgia; and it shall be the duty of all health officers, and Boards of Health in the State, to communicate to said State Board of Health copies of all their reports and publications; also such sanitary information as may be useful; and said Board shall keep record of all its acts and proceedings as a Board; and it shall promptly cause all proper information in possession of said Board to be sent to the local health authorities of any city, village or town in the State which may request the same, and shall add thereto such useful suggestions as the experience of said Board may supply; and it is hereby made the duty of said health authorities to supply like information and suggestions to said State Board of Health; and said State Board of Health is authorized to require reports and information (at such times, and of such facts, and generally of such nature and extent, relating to the safety of life and the promotion of health, as its by-laws or rules may provide,) from all public dispensaries, hospitals, asylums, prisons and schools, from the managers, principals and officers thereof; and from all other public institutions, their officers and managers, and from the proprietors, managers and lessees and occupants of all places of public resort in the State; but such reports and information shall only be required concerning matters or particulars in respect of which it may, in its opinion, need information for the proper discharge of its duties. Said Board shall, when requested by public authorities, or when they deem it best, advise officers of the State, county or local governments in regard to sanitary drainage, and the location, drainage, ventilation and sanitary provisions of any public institution, building or public place.

SEC. VII. *Be it further enacted*, That it shall be the duty of the State Board to give all information that may reasonably be requested concerning any threatened danger to the public health, to the health officers of the ports of Savannah, Darien, Brunswick and St. Marys, and to the Commissioners of Quarantine of said ports, and all other sanitary authorities in the State who shall give the like information to said Board; and said Board and said officers, said Qqarantine Commissioners, and said sanitary authorities shall, as far as legal and practicable, co-operate to prevent the spread of diseases, and for the protection of life and the promotion of health within the sphere of their respective duties.

SEC. VIII. *Be it further enacted,* That it shall be the duty of the State Board to have the general supervision of the State system of registration of births, marriages and deaths. The said Board shall recommend such forms and amendments of law as shall be deemed to be necessary for the thorough organization and efficiency of registration of vital statistics throughout the State. The Secretary of said Board shall be the Superintendent of Registration of Vital Statistics, as supervised by said Board. The clerical duties and safe keeping of the Bureau of Vital Statistics thus created, shall be provided for by the Comptroller General of the State, who shall also provide and furnish such apartments and stationery as said Board shall require in the discharge of its duties.

SEC. IX. *Be it further enacted,* That it shall be the duty of said Board, on or before the first Monday in December in each year, to make a report in writing to the Governor of this State, who shall lay the same before the next General Assembly thereafter, upon the vital statistics and the sanitary condition and prospects of the State; and such report shall set forth the action of said Board, of its officers and agents, and the names thereof for the past year, and may contain other useful information, and shall suggest any further legislative action or precaution deemed proper for the better protection of life and health.

SEC. X. *Be it further enacted,* That said Board shall meet at least every twelve months, and may also hold special meetings as frequently as the proper discharge of its duties shall require, the same to be convened by order of the President, and the rules or by-laws shall provide for the giving of proper notice of all such meetings to the members of the Board.

SEC. XI. *Be it further enacted,* That all physicians in the practice of medicine in this State shall be required, under penalty of ten dollars, to be recovered in any Court of competent jurisdiction in the State, at the suit of the Ordinary, (the amount so recovered to be appropriated to a special fund for the carrying out of the objects of this law,) to report to the Ordinary, in the forms to be provided, all deaths and births which may come under his supervision, with a certificate of the cause of death, etc.

SEC. XII. *Be it further enacted,* That where any birth or death shall take place, no physician being in attendance, the same shall be reported to the Ordinary, with the supposed cause of death, by the parents, or, if none, by the next of kin, under penalty of ten dollars, at the suit of the Ordinary, as provided in section 11 of this Act.

SEC. XIII. *Be it further enacted,* That the Coroners of the several counties shall be required to report to the Ordinary all cases of deaths which may come under their supervision, with the cause and mode of death, etc., under the penalty of ten dollars, the same to be recovered, and the proceeds applied as in section 11 of this Act.

SEC. XIV. *Be it further enacted,* That the Ordinaries of the several counties in the State shall be required to keep separate books to record all births, marriages and deaths which may take place in their counties. Said books shall always be open to the inspection of the public without fee. And the said Ordinaries shall be required to render a full and complete report of all such births, marriages and deaths to the Secretary of the said Board of Health, at such time or times as the said Board may direct.

SEC. XV. *Be it further enacted,* That it shall be the duty of the said Board of Health to prepare such forms for reports of deaths and births as they may deem proper, the said forms to be furnished by the Secretary of said Board to the Ordinaries of the several counties, whose duty it shall be to distribute them to such persons as are required by this Act to make such reports.

SEC. XVI. *Be it further enacted,* That the sum of fifteen hundred dollars is hereby appropriated for the purposes of this Act, to be drawn by the warrant of the Governor, as occasion may require, upon proper certificate and vouchers of the said Board.

SEC. XVII. *Be it further enacted,* That nothing in this Act contained shall be construed to be in conflict with the two existing Medical Boards of the State of Georgia.

SEC. XVIII. Repeals conflicting laws.

Approved February 25, 1875.

